# Teenagers and food allergy education: a systematic review

**DOI:** 10.1186/1710-1492-10-S2-A49

**Published:** 2014-12-18

**Authors:** Claire R Unruh, Cathy A Gillespie, Nancy L Ross, Allan B Becker

**Affiliations:** 1 Pediatric Allergy and Immunology, University of Manitoba, Canada; 2 Children’s Allergy and Asthma Education Centre, Winnipeg, Manitoba, Canada

## Background

As adolescents transition away from the comforts of their homes and parental guidance and into a more independent and changing lifestyle, those who are food allergic also become part of a high at-risk population for severe anaphylaxis.

Many fatal and near fatal anaphylaxis due to foods occur in young adults and teens and this age group has a great need for food allergy education as they encounter themselves developing hypothetical-deductive logic, risk-taking and greater self-consciousness.

## Methods

Systematic literature review and analysis of 142 articles. Terms included in the search included; Teens, Teen Learning Styles, Teens and Allergies/Asthma, Teens and Technology, Teens and Social Media.

## Results

It is important to understand that the most common themes found in the literature concerning teens and food allergies were very closely tied to changes that teens face in their transition to independence.

Understand that teens are constantly changing and as such, their critical thinking is developing, affecting their decision-making. The ego-centrism that develops in many teens includes the feelings that they believe they are immune to harm and less vulnerable to negative events that threaten their safety as they take risks, especially those with food allergies.

Deviations from the norm pose a threat to adolescent development and can harm teens’ psychological well-being and even their lives. To save face and preserve this norm, adolescents with food allergies are more likely to take risks (including with their allergies) and increase the potential to cause harm to themselves, especially when with peers.

Teen learning styles are also changing and now include much more technology and internet-oriented learning. Internet usage is high in teens, and they are very comfortable with cell phones and smart phones. With the help of text messages, apps, and social media networking, teens are able to make many different connections around them.

## Conclusion

This literature review showed that transition through adolescence to early adult life is a crucial time to equip teens with an understanding of how to handle food allergies in a safe manner. Expanding teen’s knowledge and establishing safe behaviors regarding food allergies is important when approaching to develop a teen food allergy education program.

Planning a program that interests, engages and educates teens about their allergies and aids them to become advocates for themselves and others with food allergies may be a crucial part in keeping this age group out of harm’s way.

